# *PAPPA2* c.392G>C Heterozygous Mutation Associates Primary Open‐Angle Glaucoma in a Chinese Family

**DOI:** 10.1155/humu/5591097

**Published:** 2026-04-15

**Authors:** Gang Wang, Zilu Guo, Jing Ren, Ge Zhang, Hanlin Tao, Yihan Liu, Siming Zheng, Xuan Zhang, Di Wang, Rumeng Zhao, Huiling Cui, Shichao Zhu, Hongen Xu, Shichao Duan, Haijun Li

**Affiliations:** ^1^ Henan Provincial People’s Hospital, Henan Eye Hospital, Henan Eye Institute, Zhengzhou University People’s Hospital, Henan University People’s Hospital, Zhengzhou, China, hnsrmyy.net; ^2^ Department of Cardiology, The First Affiliated Hospital of Zhengzhou University, Zhengzhou, Henan, China, zzu.edu.cn; ^3^ Department of Hematology, The Affiliated Cancer Hospital of Zhengzhou University & Henan Cancer Hospital, Zhengzhou, China, zzu.edu.cn; ^4^ Department of Pharmacy, Chongqing General Hospital, Chongqing University, Chongqing, China, cqu.edu.cn; ^5^ Academy of Medical Science, Tianjian Laboratory of Advanced Biomedical Sciences, Zhengzhou University, Zhengzhou, China, zzu.edu.cn

**Keywords:** fibrosis, glaucoma, IGFBP5, PAPPA2, trabecular meshwork

## Abstract

Approximately 60% of glaucoma patients have a family history. Family‐based studies indicate that relatives of glaucoma patients have a 10‐fold higher risk of developing glaucoma compared to the control population. Genetic mutations have been reported as potential contributing factors to pathogenesis of glaucoma. In this study, we reported a Chinese family that many members affected with primary open‐angle glaucoma carried a *PAPPA2* c.392G>C heterozygous mutation. Western blotting results showed that this mutation decreased PAPPA2 protein level. Strikingly, we found PAPPA2 and its substrate IGFBP5 both expressed in human aqueous humor samples, and PAPPA2 levels significantly decreased in the POAG group accompanied with IGFBP5 levels increased in the POAG group. We also demonstrated that PAPPA2 cleaved IGFBP5 in vitro. We found that overexpressed IGFBP5 increased many fibrosis‐related gene expression through mRNA sequencing, western blotting, and immunofluorescence staining assays in primary human trabecular meshwork cells (HTMCs). More importantly, we found an inadequate dose of Pappa2 caused POAG‐like phenotypes in the mouse model. So, we proved the vital function of the eye local PAPPA2‐IGFBP5 axis in regulating extracellular matrix homeostasis and contributing to trabecular meshwork fibrosis and pathogenesis of POAG.

## 1. Introduction

Glaucoma is the first leading cause of irreversible blindness worldwide, clinically characterized by irreversible optic neuropathy, with elevated intraocular pressure (IOP) being a major risk factor. Globally, approximately 3.5% of the population is affected by glaucoma, among whom about 5.7 million people experience visual field impairment and 3.1 million suffer from irreversible blindness. There will be 110 million glaucoma patients worldwide by 2040 [1–3]. Primary open‐angle glaucoma (POAG), the most common form of glaucoma, is characterized by an open anterior chamber angle and elevated or normal IOP, and its pathogenic mechanisms remain unclear [4].

Aqueous humor in the eye is produced by the ciliary body and mainly drains through the trabecular meshwork. The flow of aqueous humor maintains IOP, and trabecular meshwork fibrosis decreases aqueous humor outflow and causes elevated IOP, which is a significant risk factor for glaucoma [5–8]. Reducing IOP is the primary approach for preventing and treating glaucoma [9]. Antifibrosis of trabecular meshwork is a promising therapeutic strategy for POAG [10, 11]. Glaucoma needs more useful and novel therapeutic methods [12, 13].

On the other hand, the aqueous humor is protein‐rich and contains certain small biochemical molecules and peptides; previous studies suggest that disturbances in the local endocrine system of the eye contribute to the pathogenesis and progression of glaucoma [14–16].

Genetic mutations have been reported as potential contributing factors to the pathogenesis of glaucoma. Approximately 60% of glaucoma patients have a family history. Family‐based studies indicate that relatives of glaucoma patients have a 10‐fold higher risk of developing glaucoma compared to the control population [3]. Current genetic analyses of POAG families have revealed many cases of monogenic variants leading to POAG, such as myocilin (MYOC); optineurin (OPTN); WD repeat domain 36 (WDR36); cytochrome P450, family 1, subfamily B, polypeptide 1 (CYP1B1); and neurotrophin 4 (NTF4) [17–21].

In this study, we reported a family with several members affected by POAG; the youngest patient was 15 years old. We collected venous blood samples and isolate DNA from white blood cells. We found a *PAPPA2* c.392G>C (NM_020318.3) heterozygous mutation that was just found in affected members through whole exome sequencing and confirmed with the Sanger sequencing; this mutation caused a p.Gly131Ala alteration in the amino acid sequence. We constructed plasmids carrying wild‐type or mutant *PAPPA2* and transfected these two plasmids into 293T cells; we found that this mutation decreased the PAPPA2 protein level in the cell cytoplasm and cell culture medium supernatant. Strikingly, we found PAPPA2 and its substrate IGFBP5 both expressed in human aqueous humor samples, and PAPPA2 levels significantly decreased in the POAG group accompanied with IGFBP5 levels increased in the POAG group. We also demonstrated that PAPPA2 cleaved IGFBP5 in vitro. To figure out how the PAPPA2‐IGFBP5 axis dysfunction causing trabecular meshwork defect, we overexpressed IGFBP5 in primary human trabecular meshwork cells (HTMCs) and conducted mRNA sequencing; we found that many fibrosis‐related genes were upregulated in the IGFBP5‐OE group, and extracellular matrix (ECM)–associated pathways were enriched with differently expressed genes through bioinformatics analysis. We confirmed that overexpression of IGFBP5 upregulated COL3A1, COL4A4, aSMA, and MYOC in HTMCs with western blotting and immunofluorescence staining assays. More importantly, we found an inadequate dose of Pappa2 caused POAG‐like phenotypes in the mouse model. So, we proved the vital function of the eye local PAPPA2‐IGFBP5 axis in regulating ECM homeostasis and contributing to trabecular meshwork fibrosis and pathogenesis of POAG.

## 2. Materials and Methods

### 2.1. Blood Collection, DNA Isolation, Whole Exome Sequencing, and Bioinformatics Analysis

Venous blood samples were collected from family members using EDTA‐anticoagulant tubes; then, DNA was isolated using a kit brought from SPARKeasy (AA0901‐B, China) according to the manufacturer’s recommendations. DNA samples were sent to iGeneTech Co., Ltd (Beijing, China) to conduct whole exome sequencing using AIExome Human Exome Panel V3. The method of bioinformatics analysis was described in a previous study [22]. Human blood sample collection was approved by the Medical Ethical Committee of Henan Eye Hospital (HNEECKY‐2022 (18)).

### 2.2. Sanger Sequencing

Use the purified DNA samples as templates to amplify the genome DNA containing the mutant site of *PAPPA2* c.392G>C (NM_020318.3) using PCR technology with forward primer: GCTGGACACGCAAGAAATCC, and reverse primer: GGTCTCTGGTTTGGGTTCGT. The PCR products were sent to Sangon Biotech (Shanghai, China) to conduct Sanger sequencing.

### 2.3. RNA Extraction and Quantitative PCR (qPCR) Assays

Total RNA was extracted from cells using the SPARKeasy Cell RNA Kit (SparkJade, China) according to the manufacturer’s instructions. One microgram of total RNA was used as a template for reverse transcription according to the PrimeScript RT kit (Takara, RR037A, Japan). The cDNA was then diluted and used as a template for qPCR reactions using the 2× SYBR Green qPCR Mix (SparkJade, AH0104‐B, China). Reactions were performed on the StepOnePlus Real‐Time PCR system (Applied Biosystems, United States), and the relative expression levels of the target gene mRNA were determined against the GAPDH housekeeping gene control using the 2^−*ΔΔ*Ct^ method. The primer sequences are as follows:

*GAPDH* forward: GGAGCGAGATCCCTCCAAAAT; reverse: GGCTGTTGTCATACTTCTCATGG.

*PAPPA2* forward: AGAATAAGCCTGGCGATTTTGG; reverse: GGCCTTAGGTAGTTCCCAGC.

### 2.4. Western Blotting

Methods of aqueous humor samples and cell lysate samples were described in our previous papers [6, 15]. Patient clinical information is shown in Table 1. Eye tissue protein was extracted by RIPA buffer containing protease inhibitors with a tissue grinder (Servicebio, SWE‐3D, China). Primary antibody information is as follows: PAPPA2 (Abcam, AB277446‐1001), IGFBP5 (Proteintech, 55205‐1‐AP), COL3A1 (Abways, CY10299), COL4A4 (Proteintech, 19674‐1‐AP), aSMA (Proteintech, 14395‐1‐AP), MYOC (Proteintech, 14238‐1‐AP), and GAPDH (Proteintech, 10494‐1‐AP).

**Table 1:** Demographic features of the study population.

	POAG (*n* = 6)	Con (*n* = 6)	*p*
Subgroup proportion (%)	50	50	
Mean age (SD), years	55.50 (12.08)	60.83 (10.98)	0.423^†^
Gender			1.000^‡^
Male	3 (50%)	3 (50%)	
Female	3 (50%)	3 (50%)	
Mean IOP (SD), mmHg	32.33 (5.96)	14.0 (2.61)	~0.001 ^∗^

Abbreviations: Con = cataract; POAG = primary open angle glaucoma; SD = standard deviation.

^†^No significant difference: *p* = 0.423 for POAG versus Con (Wilcoxon rank sum test).

^‡^No significant difference: *p* = 1.000 for POAG versus Con (chi‐square test).

∗Significant pairwise comparisons: *p* < 0.05 for POAG versus Con (unpaired *t*‐test).

### 2.5. Protein Purification and Coomassie Blue Staining

Human *PAPPA2* CDS (NM_020318.3) and human *IGFBP5* CDS (NM_000599.4) were synthesized by General Biol (Chuzhou, Anhui Province, China) and constructed pCDN3.1‐Myc‐PAPPA2 and pCDN3.1‐flag‐IGFBP5 plasmids. These two plasmids were transfected into 393T cells with Lipo2000, respectively; cells were lysed in RIPA buffer containing protease inhibitors; then, myc‐PAPPA2 and flag‐IGFBP5 were purified with anti‐myc beads and antiflag beads, respectively. After incubation, beads were washed with RIPA buffer, and myc‐PAPPA2 and flag‐IGFBP5 were eluted with 1 mg/mL myc peptide and 1 mg/mL flag peptide. Protein concentrations were quantified with BSA using Coomassie blue staining buffer (MXD075, Kermey Biotech, China).

### 2.6. Cell Culture

HEK293T cells (Research Resource Identifier [RRID] CVCL_0063) were cultured in high‐glucose DMEM containing 10% fetal bovine serum, 100 U/mL penicillin, and 100 mg/mL streptomycin. The HEK293T cells used in this study were confirmed not to be contaminated with other cells and mycoplasma.

Two primary HTMC lines used in this study were isolated from two trabecular meshwork tissues which were from two different man Chinese patients’ eyes in 2022, and the isolation method was as described in our previous papers [6, 15] and cultured in DMEM/F12 medium (CD0001, SparkJade, China) supplemented with 15% fetal bovine serum, 100 U/mL penicillin, and 100 mg/mL streptomycin. Experiments were performed on primary HTMCs with no more than seven passages. Human eye collection was approved by the Medical Ethical Committee of Henan Eye Hospital (HNEECKY‐2022 (18)). Two primary HTMC lines were confirmed free of mycoplasma contamination before experiments.

### 2.7. Transcriptome Sequencing

Primary HTMCs were transfected with lentivirus carrying IGFBP5 CDS, or carrying empty vectors, and cells were collected and isolated total RNA and then constructed library for transcriptome sequencing; the detailed method was described in our previous paper [6]. Differentially regulated genes were assigned by applying thresholds of log2FC ≥ 1.5 and log2FC ≤ −1.5 with *p* < 0.05 and presented as a volcano plot.

### 2.8. Immunofluorescence Staining

Primary HTMCs were cultured on glass coverslips before applying the indicated treatments and then washed with phosphate buffered saline (PBS) before 15‐min fixation with 4% paraformaldehyde (PFA) in PBS solution at room temperature. Thereafter, the samples were permeabilized with 0.5% Triton X‐100 in PBS for 15 min, further washed with PBS before blocking with 4% BSA in PBS (0.22 *μ*m filtered) for 60 min at room temperature. The coverslips were then successively incubated with diluted primary antibodies overnight at 4°C and secondary antibodies (donkey anti‐Rabbit 488, Jackson ImmunoResearch Inc., 711‐545‐152, United States) for 60 min at room temperature, both diluted in 4% BSA in PBS (0.22 *μ*m filtered). The primary antibodies used included COL3A1 (Abways, CY10299), COL4A4 (Proteintech, 19674‐1‐AP), and aSMA (Proteintech, 14395‐1‐AP).

### 2.9. Mouse Breeding and IOP Measurement

*Pappa2* knockout mice were brought from Cyagen (Strain S‐KO‐06984, China). Female heterozygotes (*Pappa2^+/−^*) crossed with male heterozygotes (*Pappa2^+/−^*) to get homozygous (*Pappa2^−/−^*). When mice were 8 weeks old, IOPs were measured consecutively at 10 a.m. with three repeats using the TonoLab instrument with the mean value of three repeats used for analysis. Animal experiments complied with the Association for Research in Vision and Ophthalmology Statement. All animal experiments were approved by the Animal Ethics Committee of Zhengzhou University (ZZU‐LA20220729).

### 2.10. H&E Staining and Masson Staining

When mice were 8 weeks old, they were humanely sacrificed, and the excised eye were fixed with formaldehyde and embedded in paraffin sections. Five micrometer paraffin sections of mice eyes were stained with H&E staining kit (Beyotime, C0105S) and Masson staining kit (Solarbio, G1346). H&E staining and Masson staining were conducted in strict accordance with the manufacturer’s standardized protocol.

### 2.11. Statistical Analysis

Statistical analyses were performed with GraphPad Prism Version 7.0. All results are presented as mean ± SD of three independent experiments. The unpaired Student’s *t*‐test was used for two‐group comparisons or analysis of variance (ANOVA) with a Tukey correction factor for multiple group comparisons. *p* < 0.05 was considered statistically significant.

## 3. Results

### 3.1. A Chinese Pedigree With POAG

We found a five‐generation Chinese pedigree with POAG comprising 55 family members, including 13 affected individuals (Figure 1a). The affected individuals presented with an intermediate to advanced POAG phenotype. V1 is the proband, who is 15 years old, first found in the hospital. The mean age of onset was 45.2 ± 6.8 years. Clinical evaluations revealed characteristic glaucomatous optic neuropathy, evidenced by increased cup‐to‐disc ratios across affected members (Figure 1b,c), varying degrees of visual field defects (Figure 1d), elevated IOP, and open angle of the anterior chamber. The pedigree exhibited a typical autosomal dominant inheritance pattern.

Figure 1Clinical features of a family with POAG. (a) Family Pedigree. V1 is the proband. (b) Fundus examination images of six family members with POAG. (c) Cup‐plate ratio of six family members with POAG. (d) Visual field examination results of three family members with POAG.

**Figure:**
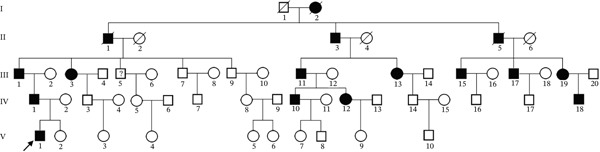
(a)

**Figure:**
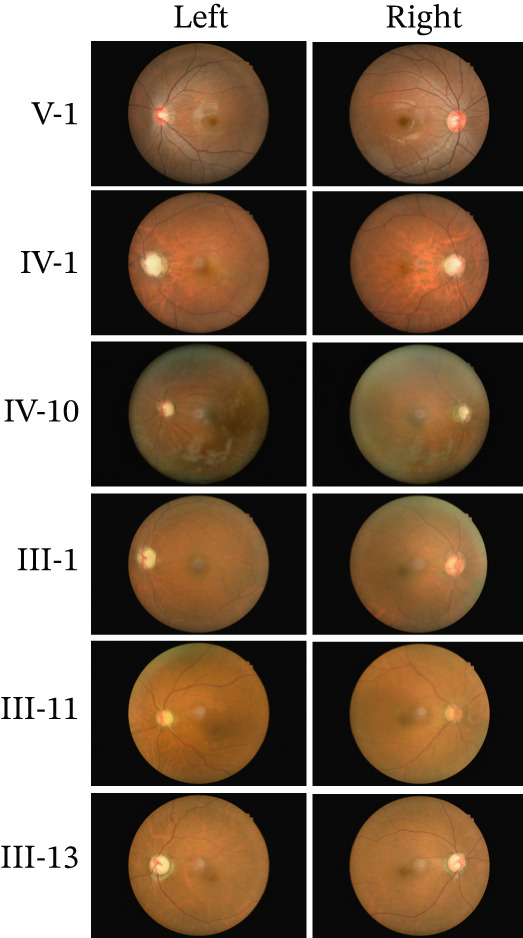
(b)

**Figure:**
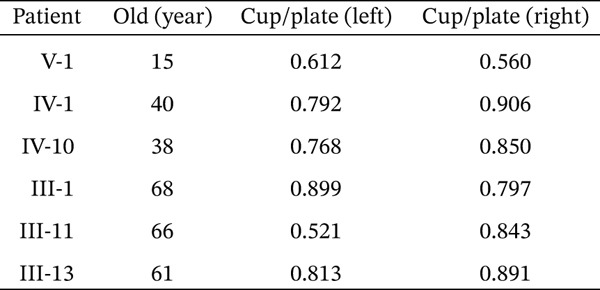
(c)

**Figure:**
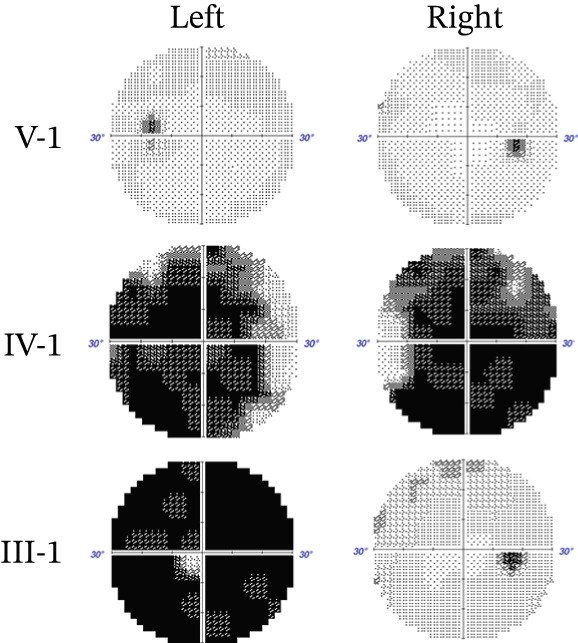
(d)

### 3.2. Whole Exome Sequencing Revealed a Candidate Variant *PAPPA2* c.392G>C in the Pedigree

To screen the mutant genes that caused POAG in this family, we collected all the living members’ venous blood samples and isolated DNA from white blood cells. Then, we sent 15 samples (10 samples from affected members and 5 samples from unaffected members) to conduct whole exome sequencing. We enriched the variant cosegregated with POAG within the family and got a *PAPPA2* c.392G>C (NM_020318.3) heterozygous mutation that just found in affected members and not found in unaffected members. Then, we confirmed this mutation site using Sanger sequencing using these 15 samples, and the results were consistent with the whole exome sequencing (Figure 2a,b). We also detected the *PAPPA2* 392G>C mutation using the other samples that did not conduct whole exome sequencing in this family; we found that the affected members all carried this heterozygous mutation, while the unaffected members did not. This mutation of *PAPPA2* was not found in the healthy population so far, so the frequency of *PAPPA2* c.392G>C in the normal population is 0.

Figure 2
*PAPPA2* c.392G>C mutation decreases its protein stability. (a) Sanger sequencing of family members with POAG indicative of *PAPPA2* c.392G>C mutation. (b) Sanger sequencing of family members without POAG indicative of wildtype *PAPPA2* c.392G. (c) Domain map of human PAPPA2. (d–f) Equal amount of pCDNA3.1‐myc‐*PAPPA2* and pCDNA3.1‐myc‐*PAPPA2* 392G>C mutant plasmids was transfected in 293T cells. qPCR assay results showed that *PAPPA2* mutant does not affect mRNA levels of PAPPA2 compared with wt. (e) Western blotting results and (f) quantification data showed that myc‐*PAPPA2* mutant decreases PAPPA2 protein level in cell cytoplasm. GAPDH served as loading control. (g, h) Cell culture medium supernatant also collected and detected myc‐PAPPA2 levels with western blotting. (g) Western blotting results and (h) quantification data showed that myc‐*PAPPA2* mutant decreases PAPPA2 protein level in cell culture medium supernatant. Ponceau staining to control the loading amount. Three independent experiments; data were presented as mean ± SD, unpaired Student’s *t*‐test.  ^∗∗^ *p* < 0.01;  ^∗∗∗^ *p* < 0.001; ns indicates no statistical significance.

**Figure:**
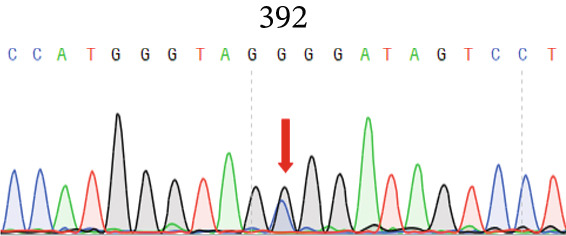
(a)

**Figure:**
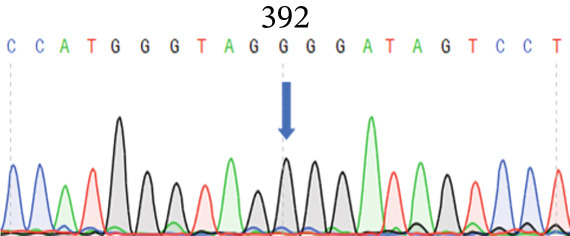
(b)

**Figure:**
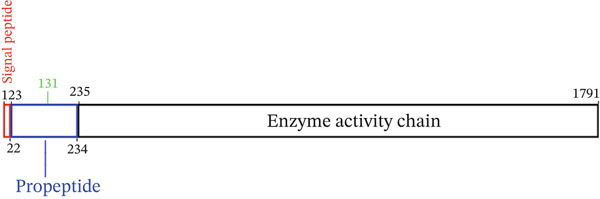
(c)

**Figure:**
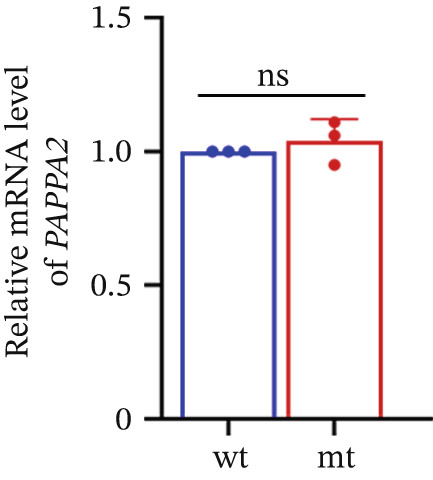
(d)

**Figure:**
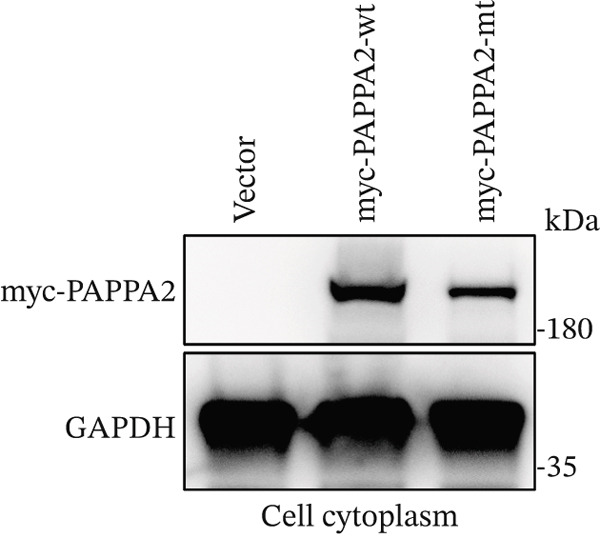
(e)

**Figure:**
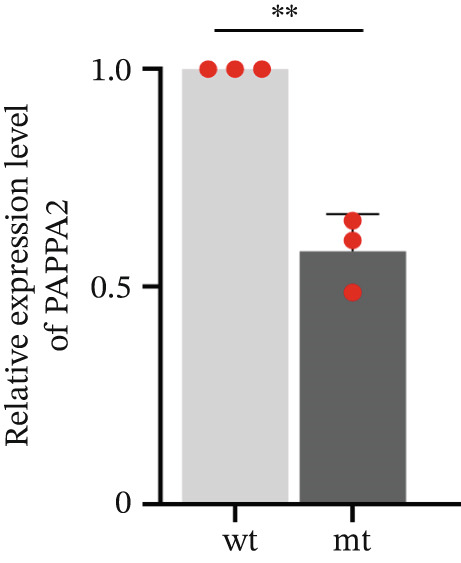
(f)

**Figure:**
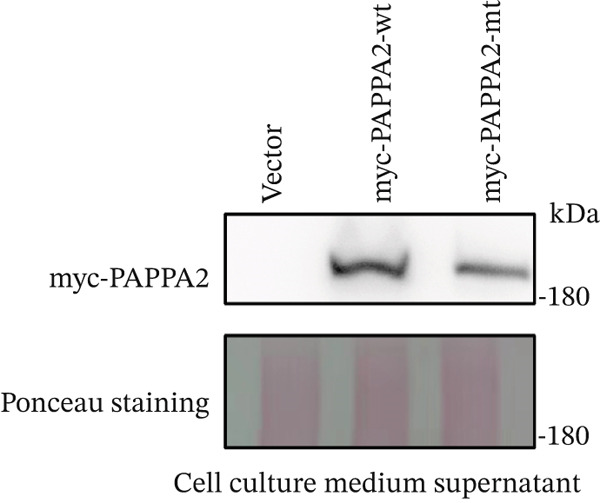
(g)

**Figure:**
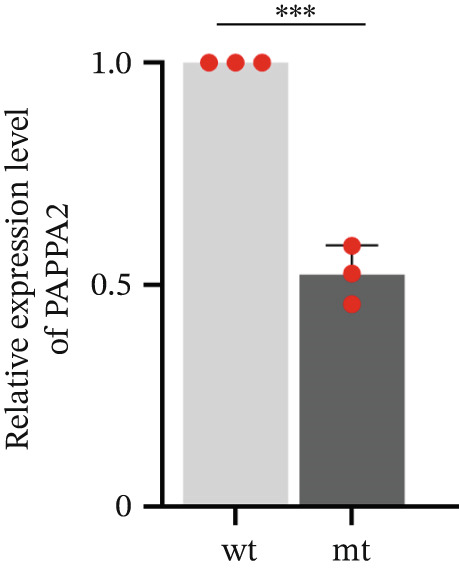
(h)

### 3.3. *PAPPA2* c.392G>C Mutation Decreases Its Protein Stability

*PAPPA2* c.392G>C mutation caused a p.Gly131Ala alteration in the amino acid sequence (Figure 2c); to detect whether this mutation affects *PAPPA2* expression, we constructed a plasmid that carried myc‐tagged wild‐type *PAPPA2* (wt) or *PAPPA2* c.392G>C (mt). Then, we transfected an equal amount of pCDNA3.1‐*PAPPA2* wt and pCDNA3.1‐*PAPPA2* mt plasmids into 293T cells. Cells and cell culture medium supernatant were collected to conduct qPCR and western blotting 48 h after transfection. qPCR assay results showed that the *PAPPA2* mutant does not affect mRNA levels of PAPPA2 compared with wt (Figure 2d). Western blotting results and quantification data showed that *PAPPA2* c.392G>C significantly decreased PAPPA2 protein levels in the cell cytoplasm (Figure 2e,f) and cell culture medium supernatant (Figure 2g,h). These data indicated that the *PAPPA2* c.392G>C mutation which caused a p.Gly131Ala alteration may affect its protein stability.

### 3.4. PAPPA2 Cleaves IGFBP5 in Eye Aqueous Humor

Based on our previous aqueous humor protein mass spectrometry data [16], we found that PAPPA2 and its substrate IGFBP5 both expressed in human eye aqueous humor. Then, we confirmed this using human aqueous humor samples with a western blotting assay (Figure 3a). More importantly, we also found lower PAPPA2 levels in POAG patients’ aqueous humor (Figure 3b), accompanied by higher IGFBP5 levels in the POAG group compared with the control group (Figure 3c).

Figure 3PAPPA2 cleaves IGFBP5 in eye aqueous humor. (a–c) PAPPA2 and IGFBP5 both expressed in aqueous humor. Western blotting results and quantification data shown (b) PAPPA2 levels are decreased in aqueous humor of POAG patients compared with control, while (c) IGFBP5 levels are increased in aqueous humor of POAG patients. Ponceau staining to control the loading amount. Coomassie blue staining images of (d) myc‐PAPPA2 and (e) flag‐IGFBP5 purified from 293T cells, BSA used to measure the protein amount. (f) PAPPA2 cleaves IGFBP5 in the EP tube at 37°C.

**Figure:**
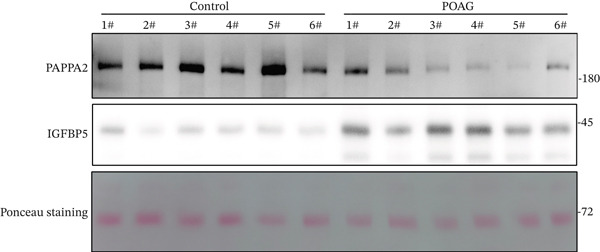
(a)

**Figure:**
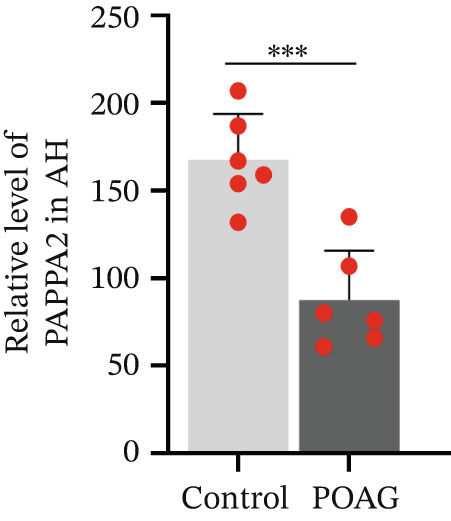
(b)

**Figure:**
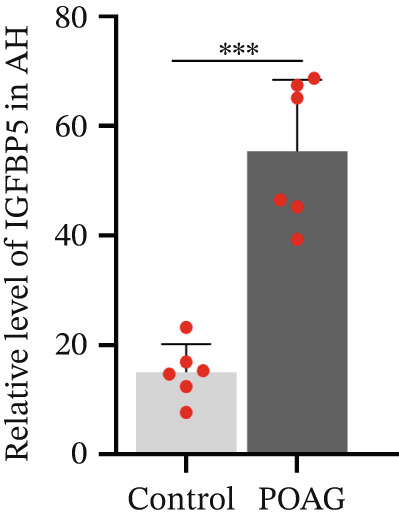
(c)

**Figure:**
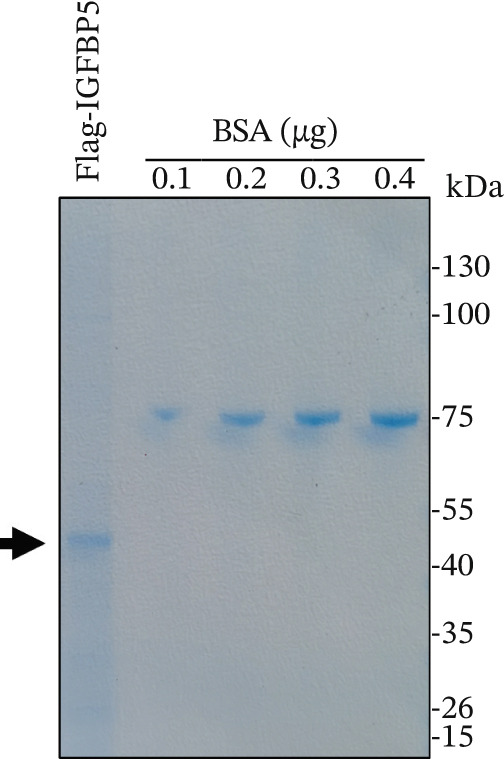
(d)

**Figure:**
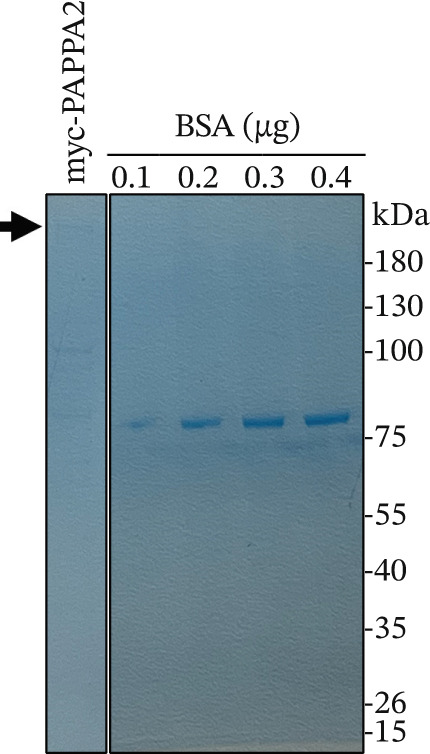
(e)

**Figure:**
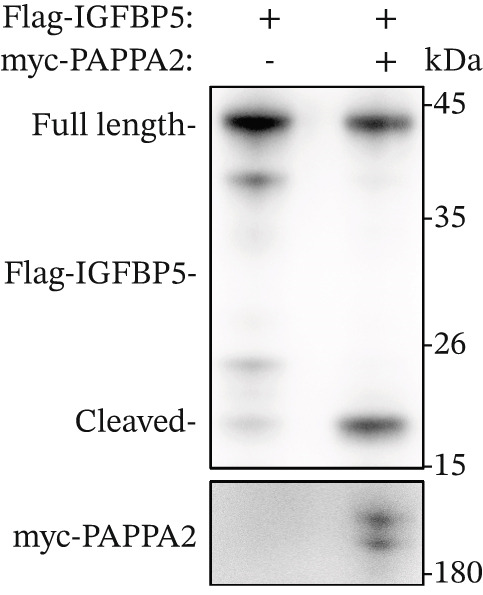
(f)

To demonstrate that IGFBP5 is a substrate of PAPPA2, we overexpressed myc‐tagged PAPPA2 and flag‐tagged IGFBP5 in 293T cells separately; then, we purified myc‐PAPPA2 and flag‐IGFBP5 with anti‐myc beads and antiflag beads, respectively. Coomassie blue staining images showed that we successfully purified myc‐PAPPA2 (Figure 3d) and flag‐IGFBP5 (Figure 3e) from 293T cells. Then, we mixed myc‐PAPPA2 and flag‐IGFBP5 in cell culture medium and incubated at 37°C for 2 h. Western blotting results showed that PAPPA2 decreased full‐length form IGFBP5, while increased cleaved form IGFBP5 compared with the control group (Figure 3f). These data proved that PAPPA2 cloud cleaves IGFBP5.

### 3.5. Overexpression of IGFBP5 Caused Transcriptome Alterations of Primary HTMCs

As *PAPPA2* c.392G>C significantly decreased PAPPA2 protein levels, which could increase its substrate IGFBP5 accumulation, similar to what we found in human aqueous humor. It is reported that IGBFP5 induces ECM production [23, 24]. To detect whether IGFBP5 could induce fibrosis of HTMCs, we overexpressed IGFBP5 in primary HTMCs using lentivirus carrying *IGFBP5* CDS and sent cells to conduct RNA‐seq to screen differentially expressed genes.

PCA image showed the IGFBP5‐OE group separated from the control group distinctly (Figure 4a). Differentially regulated genes were assigned by applying thresholds of log2FC ≥ 1.5 and log2FC ≤ −1.5 with *p* < 0.05 and presented as a volcano plot. Volcano map of transcriptome sequencing showed 2617 differently expressed genes after overexpression of IGFBP5, including 1235 upregulated genes and 1382 downregulated genes (Figure 4b). Twenty pathways were enriched with KEGG using differently expressed genes, including ECM organization and collagen metabolic process (Figure 4c). Heat map of fibrosis‐related genes showed that many collagen genes were upregulated in the IGFBP5‐OE group, including COL3A1, COL4A4, COL8A2, COL4A3, COL24A1, COL5A3, and COL11A2, while several collagen genes were downregulated in the IGFBP5‐OE group, including COL4A6, COL17A1, COL6A3, and COL13A1 (Figure 4d). IGFBP5 also upregulated CDKN1A, a cellular senescence marker gene, and MYOC, another glaucoma‐associated gene [25]. Then, on the other hand, IGFBP5 downregulated FN1 and TGFb2. These data indicated that IGFBP5 plays complicated roles in HTMCs.

Figure 4Overexpression of IGFBP5 caused transcriptome alterations of primary HTMCs. (a) Principal component analysis map of the control group and IGFBP5‐OE group. (b) Volcano map of transcriptome sequencing. Overexpression of IGFBP5 significantly upregulated 1235 genes and downregulated 1382 genes. (c) 20 pathways enriched with KEGG using differently expressed genes. (d) Heat map of fibrosis‐related genes.

**Figure:**
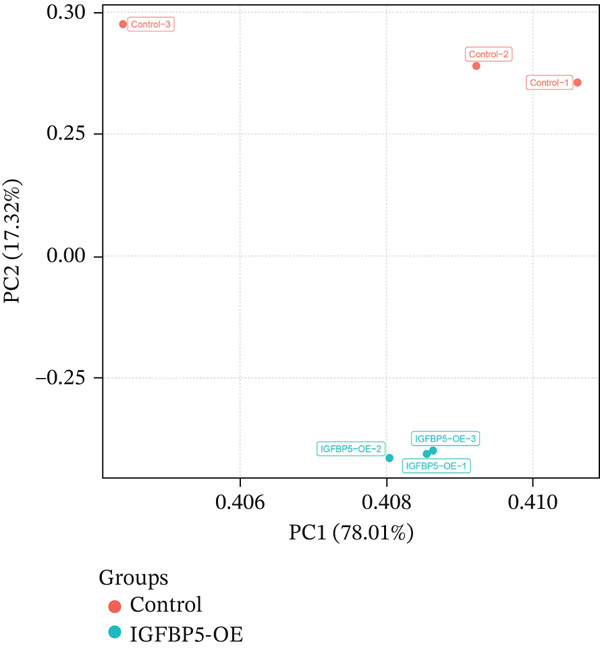
(a)

**Figure:**
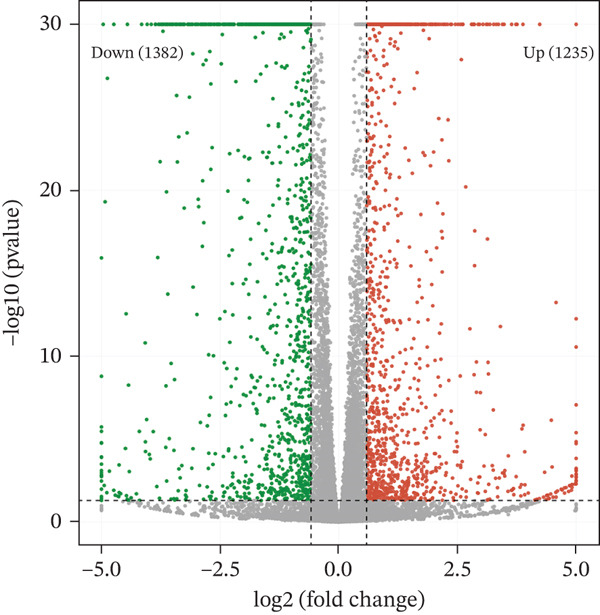
(b)

**Figure:**
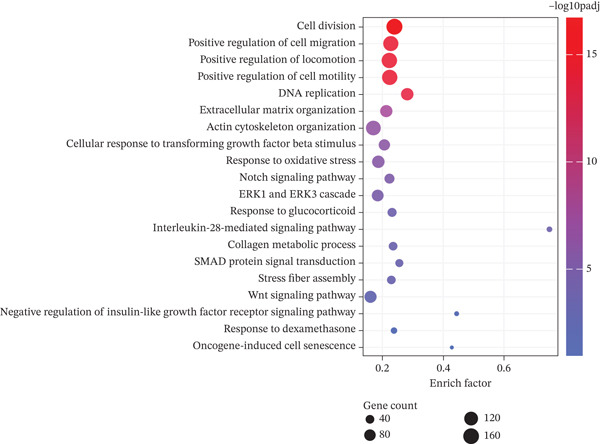
(c)

**Figure:**
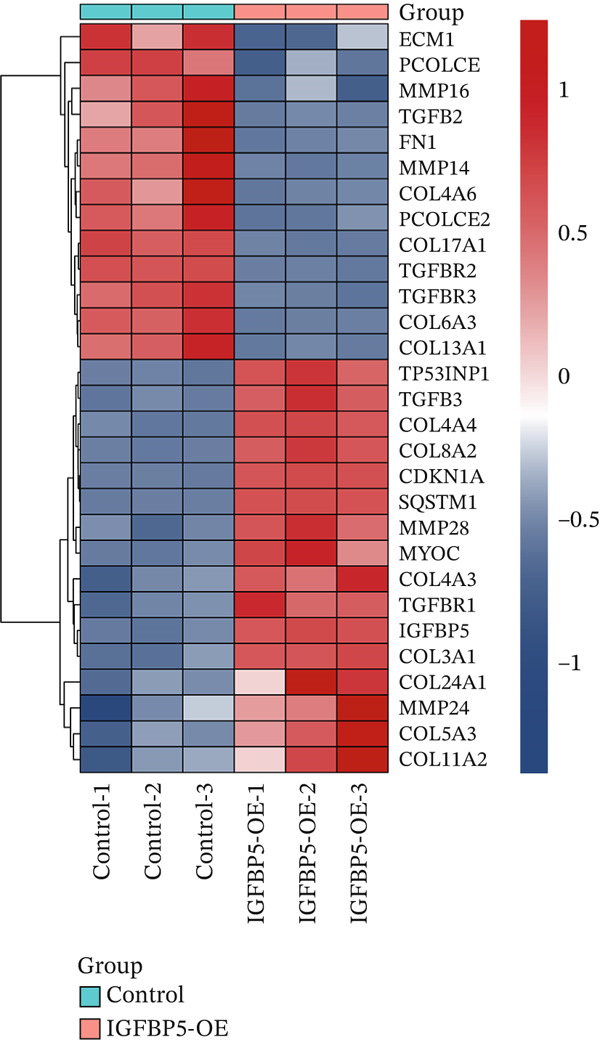
(d)

### 3.6. IGFBP5 Induces Fibrosis‐Related Proteins’ Upregulation in Primary HTMCs

Primary HTMCs were treated with 100 ng/mL IGFBP5 and 10 ng/mL PAPPA2 proteins alone or combined for 48 h, then collected cells, and conducted to western blotting assay. IGFBP5 protein treatment significantly induced fibrosis‐related protein expression, including COL3A1, COL4A4, aSMA, and MYOC in primary HTMCs‐1 (Figures 5a, 5b, 5c, 5d, and 5e) and primary HTMCs‐2 (Figures 5f, 5g, 5h, 5i, and 5j), while cotreated with PAPPA2 proteins abrogated these effects.

Figure 5IGFBP5 induces fibrosis‐related proteins’ upregulation in primary HTMCs. Two independent primary HTMCs were treated with IGFBP5, PAPPA2 proteins alone or combined for 48 h, then collected cells and conducted to western blotting assay. Western blotting and quantification results showed IGFBP5 treatment significantly upregulated (b) COL3A1, (c) COL4A4, (d) aSMA, and (e) MYOC, while PAPPA2 cotreatment decreased these proteins’ levels in (a) primary HTMCs‐1. Western blotting and quantification results showed IGFBP5 treatment significantly upregulated (g) COL3A1, (h) COL4A4, (i) aSMA, and (j) MYOC, while PAPPA2 cotreatment decreased these proteins’ levels in (f) primary HTMCs‐2. GAPDH served as loading control. Three independent experiments; data were presented as mean ± SD, analysis of variance (ANOVA).  ^∗^ *p* < 0.05;  ^∗∗^ *p* < 0.01.

**Figure:**
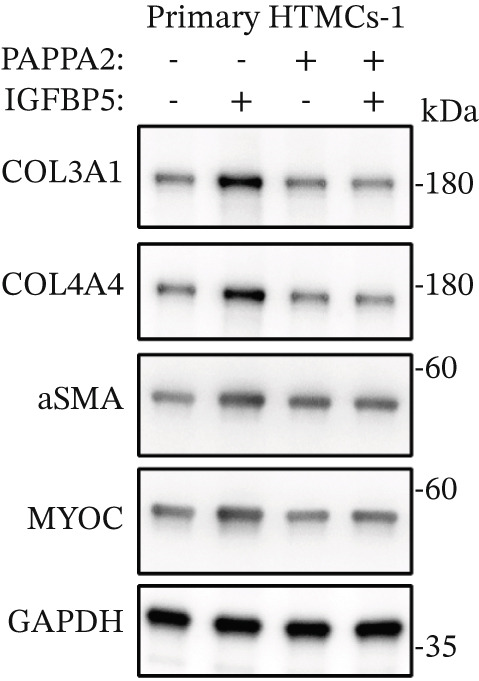
(a)

**Figure:**
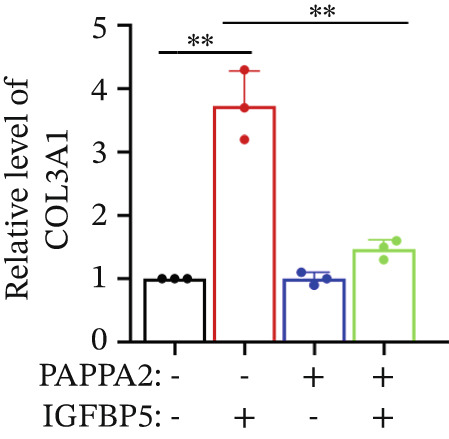
(b)

**Figure:**
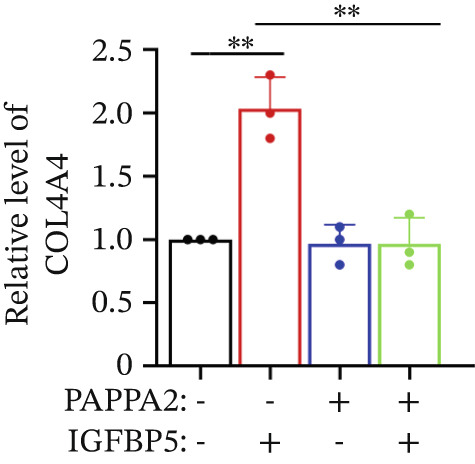
(c)

**Figure:**
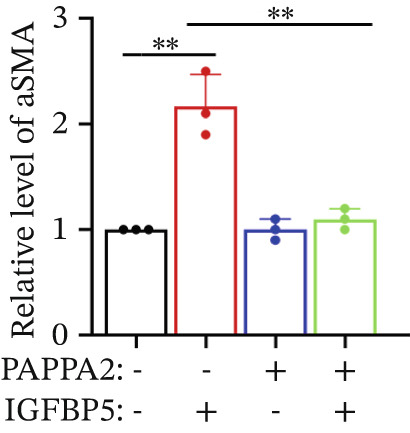
(d)

**Figure:**
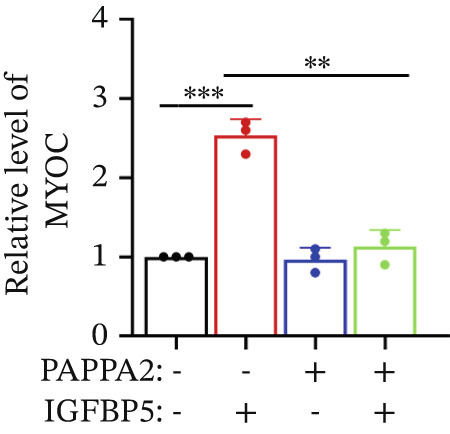
(e)

**Figure:**
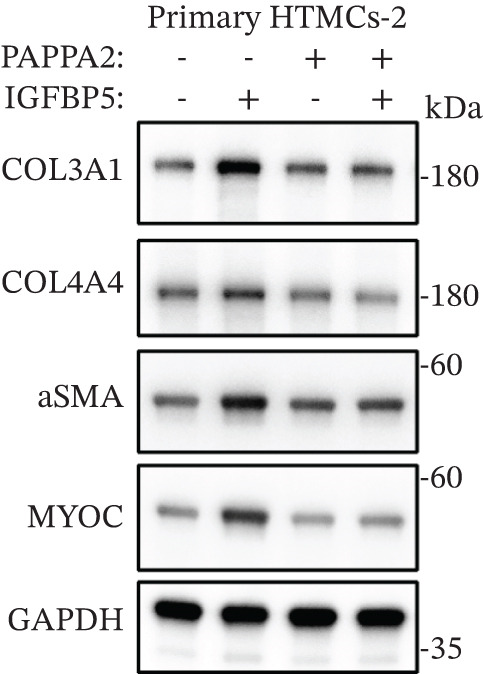
(f)

**Figure:**
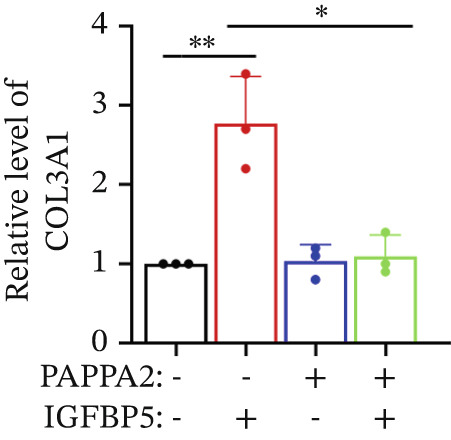
(g)

**Figure:**
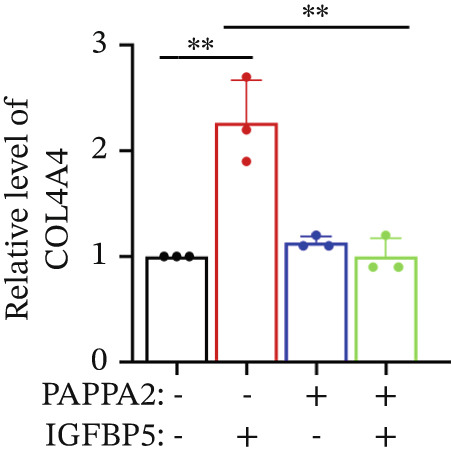
(h)

**Figure:**
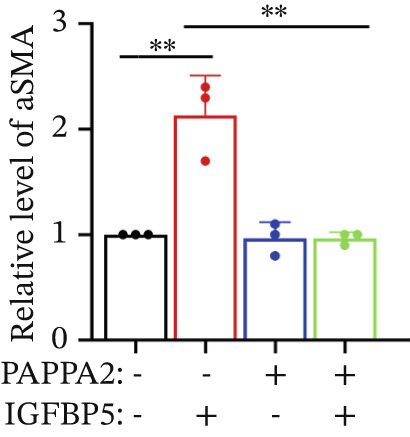
(i)

**Figure:**
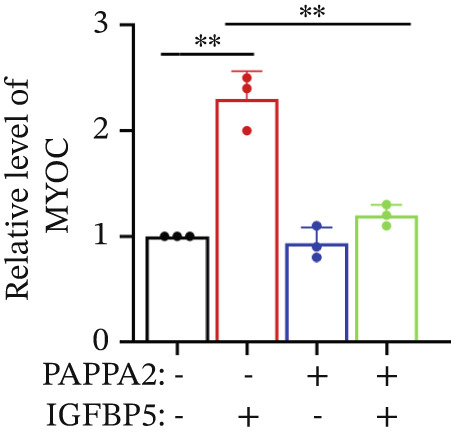
(j)

Immunofluorescence assay also found that IGFBP5 protein treatment significantly promoted COL3A1, COL4A4, and aSMA intensities in primary HTMCs‐1 (Figures 6a, 6c, 6d, and 6e) and primary HTMCs‐2 (Figures 6b, 6f, 6g, and 6h), while cotreated with PAPPA2 proteins abrogated these effects. These data indicated that PAPPA2 inhibits fibrosis‐related protein expression elevation induced by IGFBP5 treatment.

Figure 6PAPPA2 ameliorates fibrosis‐related proteins’ upregulation induced by IGFBP5 treatment in primary HTMCs. Two independent primary HTMCs were treated with IGFBP5, PAPPA2 alone, or combined for 48 h and then conducted to immunofluorescence assay. Representative images of COL3A1 (green), COL4A4 (green), and aSMA (green) of (a) primary HTMCs‐1 and (b) primary HTMCs‐2 after treated with IGFBP5, PAPPA2 alone, or combined for 48 h. Cell nucleus was stained with DAPI (blue). Fluorescence intensities of (c) COL3A1, (d) COL4A4, and (e) aSMA of primary HTMCs‐1 were quantified. Fluorescence intensities of (f) COL3A1, (g) COL4A4, and (h) aSMA of primary HTMCs‐2 were quantified. Three independent experiments, *n* = 20 cells/group per time for immunofluorescence staining assay. Data were presented as mean ± SD, analysis of variance (ANOVA).  ^∗∗∗^ *p* < 0.001.

**Figure:**
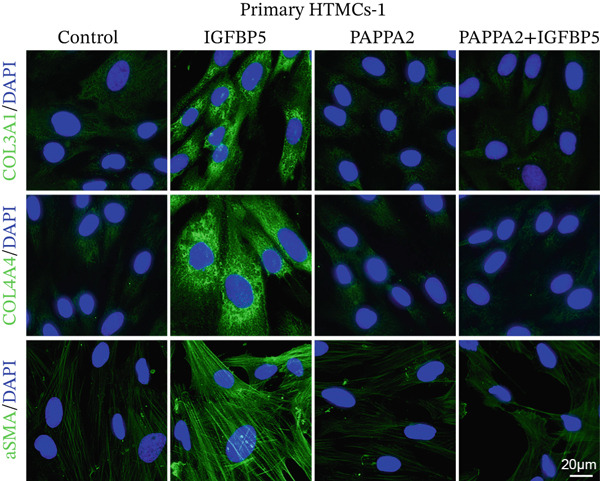
(a)

**Figure:**
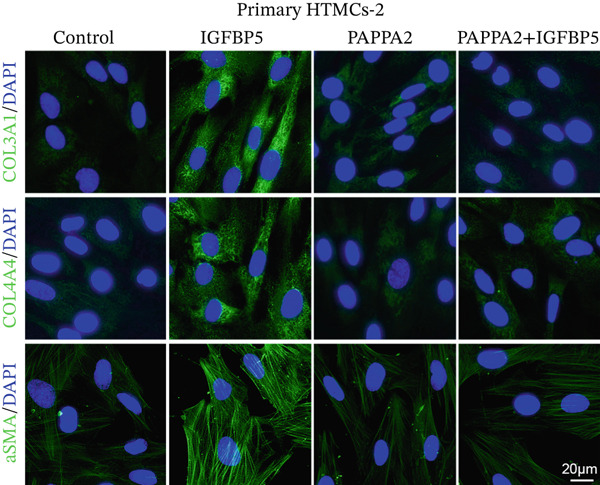
(b)

**Figure:**
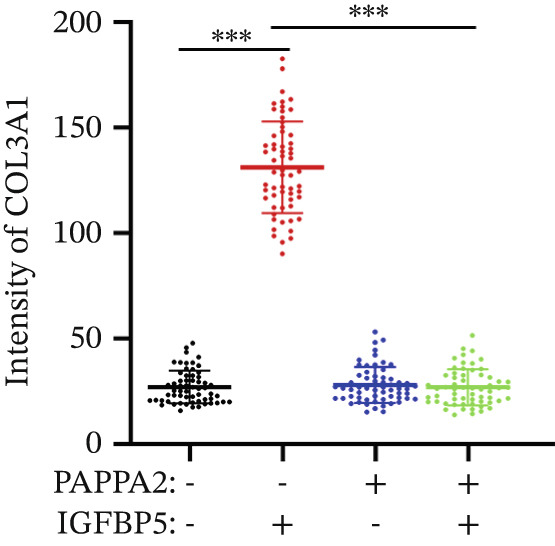
(c)

**Figure:**
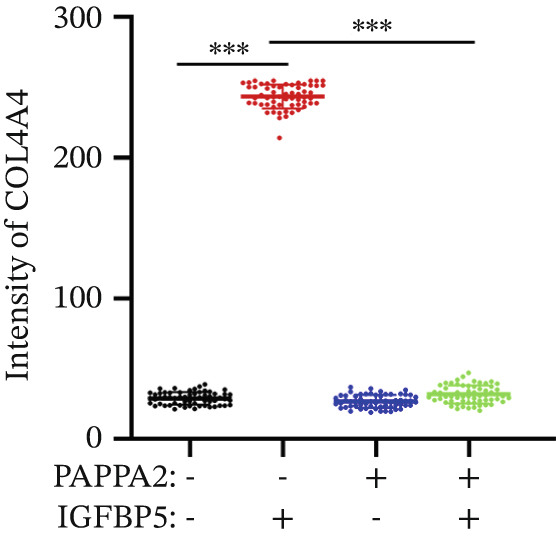
(d)

**Figure:**
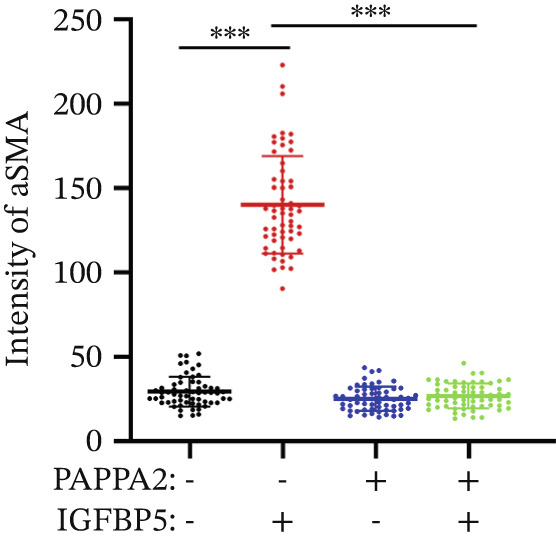
(e)

**Figure:**
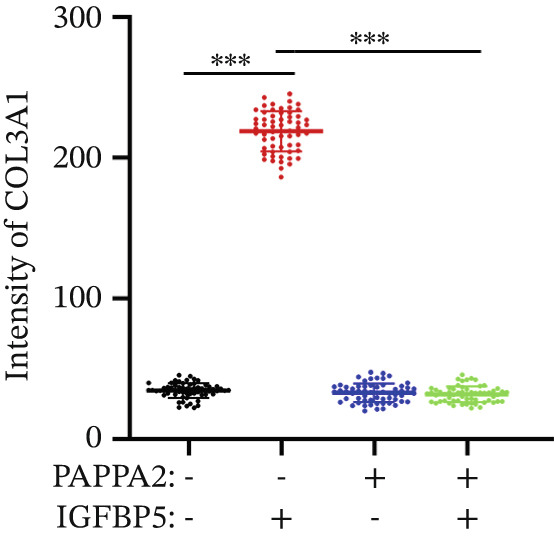
(f)

**Figure:**
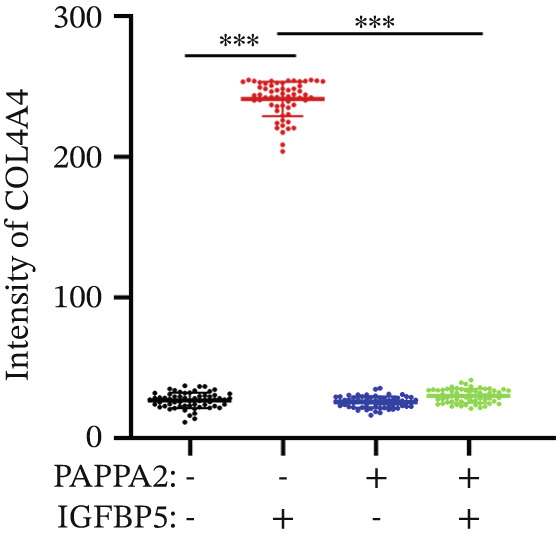
(g)

**Figure:**
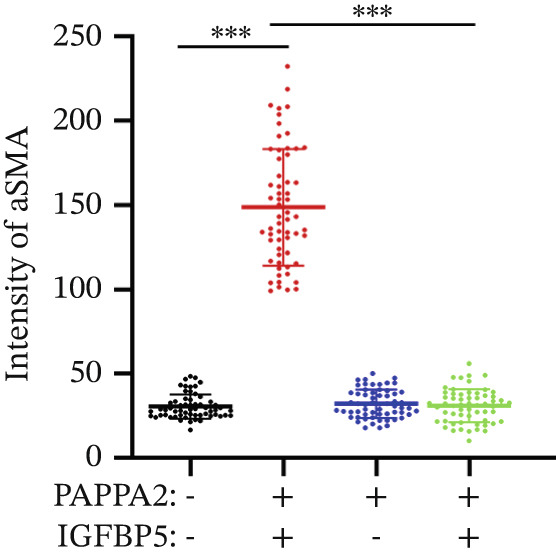
(h)

### 3.7. Inadequate Dose of Pappa2 Causes POAG‐Like Phenotypes in Mouse Model

The above data found a Chinese family carrying a heterozygous *PAPPA2* c.392G>C mutation that encodes a mutant PAPPA2 protein (p.Gly131Ala). This mutant PAPPA2 decreased its stability, which might cause IGFBP5 accumulation and induce fibrosis of primary HTMCs. We also found PAPPA2 levels decreased in the aqueous humor of POAG patients, accompanied by IGFBP5 levels in the POAG group compared with the control group. Does the eye local PAPPA2‐IGFBP5 axis have the same functions in vivo? We used *Pappa2* knockout mice to verify this hypothesis.

First, we detected Pappa2 levels in the eyes of *Pappa2* ^+/+^, *Pappa2* ^+/−^, and *Pappa2* ^−/−^ mice. Western blotting results showed that the Pappa2 protein level was decreased in *Pappa2* ^+/−^ mice eyes, and the Pappa2 protein had disappeared in *Pappa2* ^−/−^ mice. We also found that IGFBP5 levels were increased in the eyes of *Pappa2* ^+/−^ and *Pappa2* ^−/−^ mice (Figure 7a), which proved that we knock out Pappa2 in *Pappa2* ^−/−^ mice and caused an inadequate dose of Pappa2 in *Pappa2* ^+/−^ mice. Then, we measured IOP of mice at 8 weeks old, the IOPs of *Pappa2* ^+/−^ and *Pappa2* ^−/−^ mice were elevated compared with *Pappa2* ^+/+^ mice, and the IOPs of *Pappa2* ^−/−^ mice were slightly higher than *Pappa2* ^+/−^ mice (Figure 7b). H&E staining images of eye sections from three genotypes of mice showed that there is no significant difference, indicating that knocking out Pappa2 did not affect eye morphology (Figure 7c). Masson staining images of eye sections from three genotypes of mice showed that area of collagen deposition in TM tissues was significantly increased in *Pappa2* ^+/−^ and *Pappa2* ^−/−^ mice, and collagen deposition in TM tissues of *Pappa2* ^−/−^ mice was higher than in *Pappa2* ^+/−^ mice (Figure 7d,e). These data found that an inadequate dose of Pappa2 causes mouse IOP elevation, accompanied by collagen deposition in TM tissues, which proved that eye local PAPPA2‐IGFBP5 axis has significant functions in the aqueous humor flow out pathway and contributes to the pathological process of POAG.

Figure 7Inadequate dose of Pappa2 causes POAG‐like phenotypes in the mouse model. (a) Pappa2 and Igfbp5 levels in 8 weeks mouse eyes of three genotypes were detected by western blotting. GAPDH served as loading control. (b) IOPs of three genotype of mouse were measured. (c) Representative images of H&E staining of whole mouse eyes from three genotypes. (d, e) Representative images of Masson staining using eye sections from three genotypes and percentage of positive staining area in TM tissues were quantified. Every group had five mice. Data were presented as mean ± SD, analysis of variance (ANOVA).  ^∗^ *p* < 0.05;  ^∗∗^ *p* < 0.01;  ^∗∗∗^ *p* < 0.001.

**Figure:**
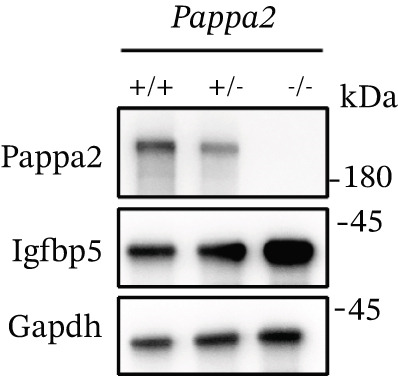
(a)

**Figure:**
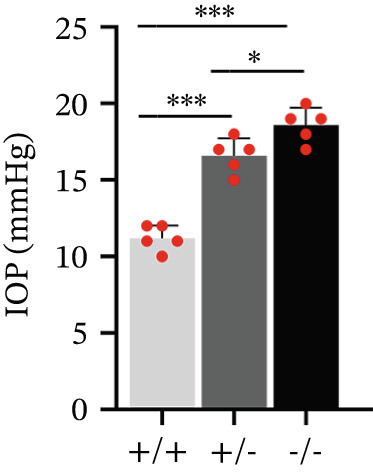
(b)

**Figure:**
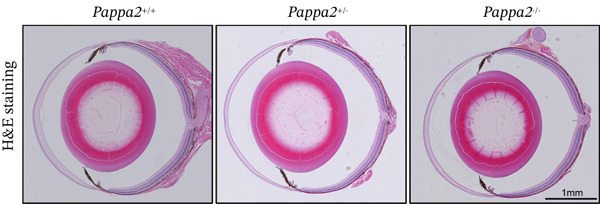
(c)

**Figure:**
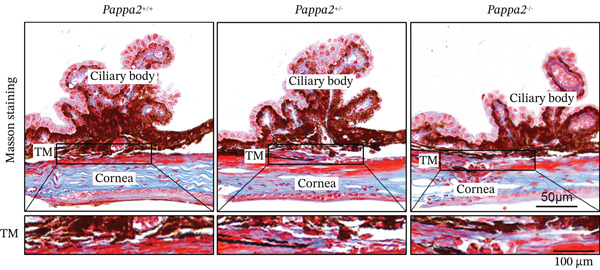
(d)

**Figure:**
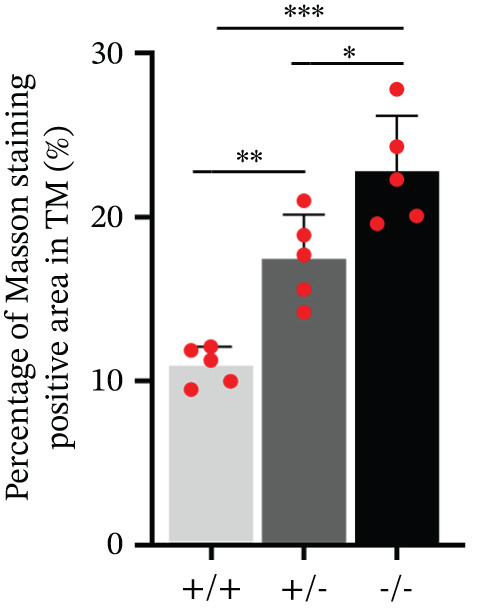
(e)

## 4. Discussion

PAPPA2 is a secretory protein belonging to the zinc metalloprotease family. It is expressed in plasma and highly expressed in placental tissue and shows low expression in tissues such as the kidney, mammary gland, brain, and pancreas. PAPPA2 primarily regulates the transport and bioavailability of insulin‐like growth factor 1 (IGF1) through the proteolytic cleavage of IGFBP5. It also exhibits partial proteolytic activity towards insulin‐like growth factor binding protein 5 (IGFBP3) and the insulin‐like growth factor‐binding protein complex acid labile subunit (IGFALS). PAPPA2 is involved in regulating glucose metabolism and skeletal development [26]. As it participates in modulating the growth hormone‐insulin‐like growth factor signaling axis, its biological functions are increasingly gaining attention [27].

The full‐length PAPPA2 protein consists of 1791 amino acids with a molecular weight of 220 kDa. Its N‐terminal 1–22 amino acids constitute a signal peptide mediating its secretion and export. Amino acids 23–234 form a propeptide, which is cleaved and released during the posttranslational folding, maturation, and activation of PAPPA2, ultimately yielding the mature and active PAPPA2 protein (a process from zymogen to active enzyme) [28].

So far, two cases carrying PAPPA2 gene variants have been reported in the previous paper; one patient carries a homozygous frameshift mutation PAPPA2 (c.1927_1928insAT, p.D643fs25∗), presenting with mild dysmorphic features including micrognathia, microcephaly, and long fingers and toes. The other patient carries a homozygous missense mutation PAPPA2 (c.3098C>T, p.A1033V), exhibiting severe postnatal growth retardation and short stature. The pathogenic mechanism in both cases may be related to elevated blood concentrations of IGF1 and IGFBP3 [29]. Studies in transgenic mouse models have shown that systemic knockout of Pappa2 results in shortened body length, reduced weight, and glucose and insulin intolerance, indicating that Pappa2 deficiency causes abnormal glucose metabolism, skeletal development abnormalities, and growth impairment in mice, resembling the phenotypes of human patients with PAPPA2 mutations. Furthermore, mice with haploinsufficiency (loss of one allele) of Pappa2 also display abnormalities in body length and weight, showing reductions compared to wild‐type controls, albeit with a milder phenotype than the full knockout mice [30, 31]. Homozygous knock‐in mouse modeling the human PAPPA2 (p.V1034A) variant exhibit phenotypes similar to the Pappa2 knockout mice [32].

In this study, we identified a missense mutation in PAPPA2 associated with a glaucoma phenotype that follows a dominant inheritance pattern, meaning individuals carrying a heterozygous mutation are affected (Figure 1). This differs from the inheritance patterns of the previously reported PAPPA2 variants. Notably, the missense mutation reported in this study is located within the propeptide region; consequently, the mature, active PAPPA2 protein does not contain this missense change. We hypothesized that the pathogenicity of this missense mutation may lie in affecting the posttranslational modification (specifically, the propeptide cleavage process) and maturation of PAPPA2. Given that the PAPPA2 propeptide comprises 211 amino acids, constituting the basis for a functional protein, it is unclear whether this propeptide has independent biological functions and whether the novel PAPPA2 mutation reported in this study acts through the propeptide as an independent functional entity to cause disease.

In our previous study, proteomic analysis of aqueous humor from patients with open‐angle glaucoma identified 1871 proteins [16], including PAPPA2, IGFBP3, IGFBP5, and IGFALS. This suggests that PAPPA2 likely plays significant functions locally within the eye. Notably, several patients from this pedigree exhibited a good prognosis following trabeculectomy, indicating that the underlying cause of the disease in this family might be dysfunction of trabecular meshwork cells leading to impaired aqueous humor outflow.

IGFBP5, the substrate of PAPPA2, plays a critical role in regulating the availability of IGFs to their receptors and thereby regulates IGF‐mediated cellular processes including proliferation, differentiation, and apoptosis in a cell‐type‐specific manner [33, 34]. IGFBP5 also induces ECM production and deposition independently of its nuclear translocation and binding to IGFs. IGFBP5 was reported to be localized to lipid rafts in human lung fibroblasts, trafficking from the plasma membrane to the nucleus through binding with Caveolin‐1, a protein involved in membrane trafficking and signal transduction in tissue fibrosis. On the other hand, IGFBP5‐induced ECM production in vitro in primary human fibroblasts may be independent of its nuclear translocation, as reduced IGFBP5 translocation to the nucleus did not inhibit the ability of IGFBP5 to induce ECM production and a fibrotic phenotype [23, 24]. IGFBP5 can also induce the production of ROS to mediate the profibrotic process, and decreasing ROS levels could block IGFBP‐5‐stimulated ECM production [35]. It reported that IGFBP5 upregulated DOK5 mRNA and protein levels to promote fibrotic phenotypes [36]. While how IGFBP5 induces fibrosis of HTMCs, whether it shares similar pathways with other cell types, has been reported, it needs further study.

We found that PAPPA2 c.392G>C decreases the protein levels in the cell cytoplasm and cell culture medium supernatant (Figure 2). We also found that PAPPA2 and its substrate IGFBP5 both expressed in human aqueous humor samples, and PAPPA2 levels significantly decreased, and IGFBP5 levels increased in the POAG group. We also demonstrated that PAPPA2 cleaved IGFBP5 in vitro (Figure 3). We proved that overexpression of IGFBP5 upregulated COL3A1, COL4A4, aSMA, and MYOC in HTMCs with mRNA sequencing (Figure 4), western blotting (Figure 5), and immunofluorescence staining assays (Figure 6).

More importantly, we found that *Pappa2* ^+/−^ and *Pappa2* ^−/−^ exhibited POAG‐like phenotypes in a mouse model with elevated IOP and trabecular meshwork fibrosis (Figure 7). The PAPPA2 c.392G>C mutation might cause a low level of PAPPA2 in aqueous humor, subsequently increasing IGFBP5 level in aqueous humor and inducing trabecular meshwork fibrosis, which in turn leads to obstructed aqueous outflow, elevated IOP, and contributes to the pathogenesis and progression of glaucoma, while the eye local PAPPA2‐IGFBP5 axis may have other important roles, which need further study in the future.

Our collective data proved the vital function of the eye local PAPPA2‐IGFBP5 axis in regulating ECM homeostasis and contributing to trabecular meshwork fibrosis and pathogenesis of POAG.

**Nomenclature**
POAG: primary open‐angle glaucoma
IOP: intraocular pressure
WES: whole exome sequencing
PAPPA2: pappalysin 2
IGFBP5: insulin‐like growth factor binding protein 5
HTMC: human trabecular meshwork cell
TM: trabecular meshwork
COL3A1: Collagen, Type III, Alpha 1
COL4A4: Collagen, Type IV, Alpha 4
aSMA: alpha smooth muscle actin
IGF1: insulin‐like growth factor 1
IGFBP3: insulin‐like growth factor binding protein 5
qPCR: quantitative polymerase chain reaction

## Author Contributions

Haijun Li Li, Shichao Duan, and Hongen Xu conceived and designed the experiments. Hongen Xu did the bioinformatics analysis of WES. Gang Wang, Zilu Guo, Jing Ren, and Ge Zhang performed the major in vitro experiments. Yihan Liu, Hanlin Tao, Huiling Cui, Siming Zheng, Xuan Zhang, Di Wang, and Rumeng Zhao analyzed the immunofluorescence staining and western blotting data. Gang Wang and Shichao Duan preformed the mouse‐related experiments. Haijun Li, Shichao Duan, Hongen Xu, and Shichao Duan wrote the paper. Gang Wang, Zilu Guo, Jing Ren, and Ge Zhang have contributed equally to this paper.

## Funding

This work was supported by National Natural Science Foundation of China (U1904166), Henan Medical Science and Technology Research Plan (SBGJ2018072 and SBGJ202502025), Henan Province Natural Science Foundation (242300421284 and 262300422299), Henan Clinical Research‐Oriented Doctor Program (HNCRD202502), and Henan Medical Researcher Overseas Training Program (GCC2026039).

## Ethics Statement

This study was performed in line with the principles of the Declaration of Helsinki, and it was approved by the Medical Ethical Committee of Henan Eye Hospital (HNEECKY‐2022 (18)).

## Consent

Written informed consent was obtained from all the participants (for minors, guardians signed the consent).

## Conflicts of Interest

The authors declare no conflicts of interest.

## Data Availability

The data of this study are available from the corresponding authors upon reasonable request.
